# Exploring the effect of professionalization, risk-taking and technological innovation on business performance

**DOI:** 10.1371/journal.pone.0263694

**Published:** 2022-02-10

**Authors:** Francisca García-Lopera, José Manuel Santos-Jaén, Mercedes Palacios-Manzano, Daniel Ruiz-Palomo

**Affiliations:** 1 Department of Applied Economics (Mathematics), University of Malaga, Malaga, Spain; 2 Department of Accounting and Finance, University of Murcia, Murcia, Spain; 3 Department of Finance and Accounting, University of Malaga, Malaga, Spain; University of Almería, SPAIN

## Abstract

The aim of this paper is to analize the influence of professionalization over firm’s performance and the effect of two mediating variables, risk-taking and technological innovation. A total of 310 Spanish SMEs were surveyed, and the study was conducted using partial least squares path modelling (PLS-SEM) technique. The findings showed that firm’s performance is influenced by professionalization, risk-taking and technological innovation. These effects are not only direct and positive, but there are also important indirect effects that reinforce the positive effects of professionalization on firm’s performance. This research contributes to the literature on professionalization considering mediating effects of risk-taking and technological innovation in the relationship between professionalization and firm’s performance. The results provide interesting implications for theory and practice, indicating how companies can orient their strategies with the aim of gaining competitive advantage in order to increase their performance.

## 1. Introduction

In the current economic and social climate, companies, especially Small and Medium-sized Enterprises (SMEs), have to maximise their growth opportunities, so they have to direct their strategies towards seeking competitive advantages that will allow them to differentiate themselves from their competitors. This search for competitive advantages requires an adequate professionalization of the companies. As Diéguez-Soto et al. [1] have already argued, professionalization increases profitability and achieves competitive advantages for firms. Therefore, professionalization is recognized as a need for firms to be successful [2] and achieve improved financial and non-financial performance.

According to Kaplan and Norton [3], the professionalization of the company through the improvement of internal processes determines the critical organisational activities that will enable the company to improve its productivity and achieve differentiated value in order to achieve its development objectives. After successful professionalization, companies achieve professional management with higher degrees of structure and defined processes [4].

Based on an appropriate professionalisation, companies must direct their strategy towards launching new market offerings, take risks as a result of trying new products, services, or markets, and be more proactive than rivals [5]. At this point, risk-taking appears as the firm characteristic to take risks by strategically considering the business-related opportunities in uncertain situations. According to Soto-Acosta et al. [6], Technological innovation is the strategy defined for the transformation of ideas and knowledge into these new products and processes.

Previous literature is very prolific in studying the relationship of these variables to each other. However, it is not common to find studies as a whole. Thus, many studies analyse the influence of professionalization on performance [7–9] or risk-taking on innovation [10, 11]. However, the goal of this paper is to analyze how both risk-taking and technological innovation mediate the effect of professionalization on SMEs’ performance. The key research questions we are trying to answer are: Does professionalization influence performance in SMEs?; Is this relationship mediated by risk-taking or technological innovation?; Is the effect of professionalization on performance mediated by risk-taking?. With this purpose, a Structural Equations Modeling based on Partial Least Squares (PLS-SEM) has been developed in a sample of 310 Spanish SMEs. PLS-SEM is a suitable technique for solving problems even when very complex relationships exist because the optimisation algorithm maximises the explained variance of the model’s independent variables [12]. In this way, PLS-SEM has allowed us to understand the causal relationships between latent variables [13].

The findings in this paper have demonstrated the effect of professionalization on firm performance and the mediating effect of risk-taking and technological innovation on this relationship. For this reason, this paper contributes to previous research by demonstrating how SMEs can only expect to improve their performance if they first carry out an adequate professionalization process that allows them to implement a risk-oriented strategy and to implement appropriate technological innovation.

This paper continues in section 2, showing the hypotheses development. In section 3, the research methodology is established. The results obtained after applying PLS-SEM are presented in section 4. Finally, section 5 concludes with a discussion of these results and a description of the practical and theoretical implications and limitations.

## 2. Literature review

The reason for human resource professionalization is established in agency theory, which theorizes that managers will follow self-interested goals, rather than the owner’s goals, if their behavior is not monitored [14]. From an agency perspective, combined ownership and management may reduce the threat of agency problems related to information asymmetries and managerial appropriations [15].

Several studies have examined the impact of professionalization on firm performance, and in general, the results show that professionalization improves financial performance. [7–9]. Most previous research on the professionalization of business have been carried out in family firms [7, 9, 16] and Western economies [17].

Madison et al. [8] conducted research to investigate the treatment of employees in family and non-family organizations and how it affects firms’ performance. Results revealed that human resource professionalization is positively associated with the performance of the family firm. In this line, Lien and Li [16] showed that, adopting non-family management in post-IPO family firms harms firm performance due to weak governance institutions in Taiwan. However, more research on non-family employees is needed. For this reason, we establish the following hypothesis:

H_1_: *Professionalization is positively related to performance*.

The professionalization process encompasses many different aspects that a firm must address [9]; among them is the definition of the level of Risk-taking that the company is willing to undertake.

Professionalization enables the company to better understand and assess the developments around it. This will enable it to react much more quickly to changes in the environment. Therefore, the company is willing to accept larger venturing risks [18] since the company will feel more able to take risks [19]. In the same vein, a low level of professionalization in business makes it difficult to step out of one’s comfort zone and adopt Risk-taking [20].

Previous studies have shown that the more professionalized a company is, the more Risk-taking it is likely to be [20–23]. In this respect, it has been shown that a long-term orientation, through better use of resources and skills following successful professionalization, can provide a favourable context for increased entrepreneurial Risk-taking [24].

Literature has recognised Risk-taking as the central feature of entrepreneurship and a contributor to performance [25]. Based on one of the most fundamental asset pricing theories in financial economics theory, the Capital Asset Pricing Model [26], Risk-taking directly influences performance, through the risk-return trade-off since entrepreneurs will only take on the riskier or more uncertain ventures/strategies if they are accompanied by a higher expected return [25]. Moreover, Risk-taking provides companies the capabilities to transform themselves in response to changes in the environment, with the aim of obtaining a competitive advantage and ensuring long-term survival [27]. This is because a risk orientation allows companies to introduce new products and brands ahead of their competitors [28]. In the same vein, Hoskisson et al. [29], claimed that Risk-taking is vital for managers to compete in a dynamic market and to respond the competitive threats. Similarly, Putniņš and Sauka [25] demonstrated that constructive Risk-taking is the central driver of company performance, mirroring the principle of risk and return in financial investment settings. Hence, it can be argued that as firms invest in new projects, they take financial risk and greater risk conditions will result in higher financial performance [30].

Based on the above, previous studies have shown the positive effect of Risk-taking on performance [30–32].

Professionalization helps firms cope with their competitive environment, increase strategic decision-making quality, and thus increase the firm’s performance [33–35]. At the same time, risk-taking directly correlates with performance, which can be understood through the risk-return trade-off central to financial economics theory [25].

We further propose that professionalization positively contributes to performance by impacting the types of risk taken within the firm. For this reason, it is to be expected that Risk-taking mediates the relationship between professionalization and performance (a mediated relationship).

Given the above, we establish the following research hypothesis:

H_2_: *Risk-taking partially mediates the relationship between professionalization and performance*.

This H_2_ hypothesis is sub-divided into the following three hypotheses:

H_2a_: *Professionalization has a positive effect on Risk-taking*.
H_2b_: *Risk-taking has a positive effect on performance*.
H_2c_: *Professionalization indirectly affects performance through Risk-taking*.

Professionalization supports technological innovation across its initiation, adoption and implementation [1]. Therefore, technological innovation in the firm may be conditioned by the knowledge that the firm can contribute during the innovation process [36]. In this sense, Liang et al., [37] stated that the performance of technological innovation depends mostly on the process of knowledge creation. Thus, the greater the professionalization of the firm, the greater its capacity to innovate is.

According to [38], the professionalization of a company gives it advantages in terms of new product development. For example, the more professional the company’s CEO is, the greater the capacity to identify opportunities for change and of developing strategic planning and management through the innovation of products, services, or processes [39].

Moreover, according to Resources-Based View (RBV), professionalization provides firms with resources, ideas, labels and visions with which to build and develop the firm, thereby increasing the incentive to invest in innovation [40], as well as affecting the innovation capacity of the whole organisation [41]. Professionalization improves internal processes reducing cost or increasing quality and reliability [1].

Based on the above, it is expected that firm professionalization will develop better strategic planning and management through the innovation of products and processes.

The concept of innovation can be defined as new knowledge and ideas transformed into new products and/or services, new technologies, new processes and new organizational structures [42].

Technological innovation is a key factor in business performance and plays an important role in the corporation’s growth. SMEs have to monitor their competitive position through innovation [43]. It is necessary to raise competitiveness capability and intensify firm efficiency and productivity [44].

The topic of the impact of innovation on firm performance has been studied by previous researchers [11, 45–47]. Relevant studies reveal that the two concepts are positively and significantly correlated [48–51].

A firm’s knowledge capabilities, not only effectively but also innovatively, allow it to improve its performance [6, 52]. According to Darroch [52], knowledge management can be considered a synchronizing tool used to translate these assets into capabilities that enhance organizational performance. Similarly, Soto-Acosta et al. [42] argued that technological innovation provides firms with a strategic orientation to achieve sustainable competitive advantage. This explains why technological innovation has become an essential factor that contributes to business performance.

This is because companies that develop more innovative products and services achieve benefits over their competitors [50] because innovative products and services face less competition to be introduced in the market, allowing the company to increase profits and differentiate itself from the competition [53–55].

As has been established above, professionalization provides companies with greater capabilities to carry out appropriate technological innovation. This technological innovation, in turn, enables firms to gain competitive advantages that allow them to differentiate themselves from their competitors and increase their performance. It is, therefore, to be expected that an increase in the professionalization of firms will, in turn, have an indirect and positive effect on performance by increasing innovative capacity.

Given the above, we establish the following research hypothesis:

H_3_: *Technological innovation partially mediates the relationship between professionalization and performance*.

This H_3_ hypothesis is sub-divided into the following three hypotheses:

H_3a_: *Professionalization has a positive effect on Technological innovation*.
H_3b_: *Risk-taking has a positive effect on performance*.
H_3c_: *Professionalization indirectly affects performance through Technological innovation*.

The link between Risk-taking and innovation has been proven by several scholars [10, 11]. Technological innovation involves not only an initial expenditure on R&D but also uncertain benefits [25]. For its part, Risk-taking indicates the companies’ willingness to invest resources in technological innovation [56]. In the same vein, Miller and Friesen [5] suggest that Risk-taking is “entrepreneurial” when it is associated with innovation.

An adventurous entrepreneurial spirit in business without fear of risk and in search of higher returns facilitates experiments, among which are acquiring, learning and absorbing new external technology [57]. In accordance with the preceding, Mao and Zhang [58] state that Risk-taking is an important driver of firm technological innovation.

The mediating effect of innovation on the relationship between Risk-taking and performance has been studied in the literature [59]. Regarding technological innovation, the resource-based view of the firm suggests that technological innovation mediates the relationship between Risk-taking and performance [60]. Based on this theory, when resources are constrained, companies might be forced to combine the competing demands and engage in innovative Risk-taking, directing this Risk-taking towards innovative activities [25]. Therefore, this indirect approach consists of improving companies’ innovation capabilities. In this line, Jeon [61] proved that technological innovation has a mediating effect on the relationship between Risk-taking and performance.

In the same way, on the basis of the established above, the professionalization of firms can be expected to have a spill-over effect on technological innovation. This professionalization will not only provide them with the necessary knowledge with which to carry out the implementation of technological innovation successfully. However, it will also allow them to have the capacity to assume the risks that any technological innovation process entails. For this reason, it is to be expected that Risk-taking mediates the relationship between professionalization and technological innovation.

Based on the information provided above, we state the following hypotheses:

H_4_: *The effect of Risk-taking on performance is partially mediated by Technological innovation*.
H_5_: *Risk-taking partially mediates the relationship between Professionalization and Technological innovation*.

The H_4_ hypothesis is sub-divided into the following two hypotheses:

H_4a_: *Risk-taking is positively associated with Technological innovation*.
H_4b_: *Risk-taking indirectly affects performance through Technological innovation*.

On the basis of all the above, it seems reasonable to believe that by increasing professionalization, Risk-taking increases the capacity of companies to carry out innovative activities, which has an impact on their performance. Therefore, a sequential mediation of the relationship between professionalization and performance by Risk-taking and Technological innovation can be expected. Hence, we propose the following hypothesis:

H_6_: *Risk-taking and Technological innovation sequentially mediate the relationship between Professionalization and performance*.

**Fig 1:**
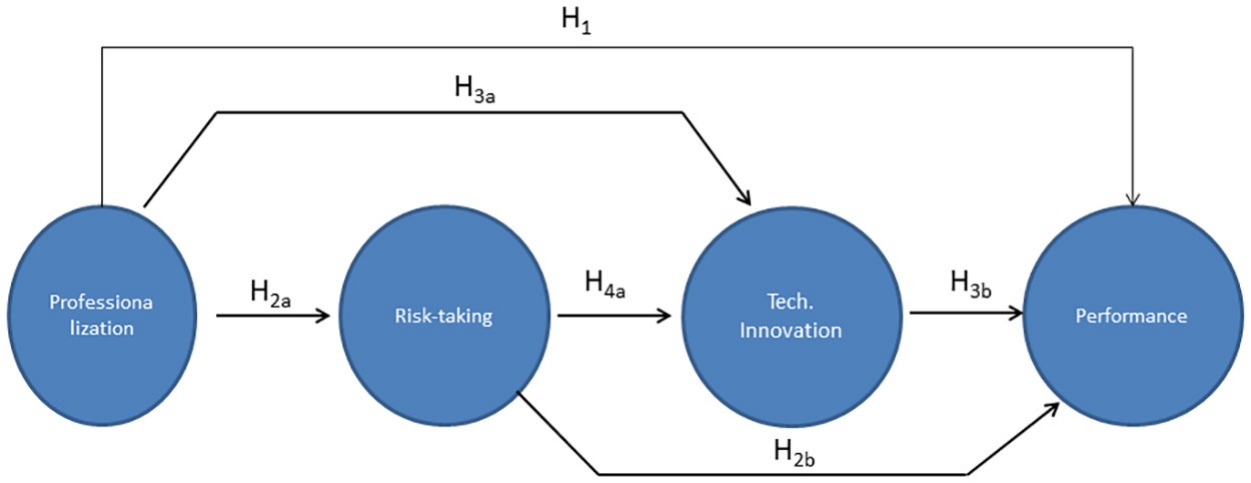
Proposed model.

In order to test the hypotheses put forward, in Fig 1 our proposed model is depicted. It shows four constructs, an exogenous construct (professionalization) and three endogenous constructs (Risk-taking, technological innovation and performance).

### 2.1. Sample

This study was carried out with a sample of 310 Spanish SMEs. For this purpose, the Central Business Directory of the National Statistical Institute (INE) was used. The sample was segmented by activity and size. The distribution of the sample is presented in Table 1. The sample was selected in SABI database through the principles of stratified sampling for finite populations. The estimation of the sample considered a relative frequency of answers in a specific item is p = 0.5, to a maximum error of 5.6% at a confidence level of 95%. To confirm that statistically significant relationships will be identified in the proposed model and that the sample size is sufficient to carry out the research. A post hoc analysis has been conducted using the G*Power Version 3.1.9.4 software tool [62]. Assuming a standard error of 0.05 and an effect size of 0.15, we have calculated the statistical power of the sample, which is 1 (over than the shortcut value of 0.8 [63]). Therefore the sample size is acceptable.

**Table 1:** Sample composition.

		**Size**(Number of employees)							
Total of companies		Micro companies (<10)		Small companies (10–49)		Medium companies (50–250)			
Number	Percent of total	Number	Percent of total	Number	Percent of total	Number	Percent of total		
310	100	111	35.80%	130	41.90%	69	11.80%		
		**Activity**							
Total of companies		Manufacturing		Construction		Trade & Commerce		Services	
Number	Percent of total	Number	Percent of total	Number	Percent of total	Number	Percent of total	Number	Percent of total
310	100	98	31.60%	90	29.00%	60	19.40%	62	20.00%

Source: Authors

The information was obtained through telephone surveys of company managers from October 2016 to January 2017. The survey was carried out personally with managers, as they are the ones who know best the objectives, strategies, concerns of the companies [64]. According to Fisher [65], to reduce social acceptance bias, the survey was anonymously undertaken. The nonresponse bias has been analysed through the t-test and the chi-squared test for all the constructs. For this purpose, the responses have been divided into two groups, a first group containing the first responses (75% of the responses) and a second group containing the last responses (25% of the responses). The results show that there are no problems concerning the nonresponse bias. Finally, a standard method bias has been discarded when analysing the results of the variance inflation factors (VIF). The results, which will be shown in the following section, show how the VIF varies from 1.525 to 2.608. Thus, all the values are smaller than 3.3., common method bias is not a problem in this research [66].

### 2.2. Measures

Based on previous studies, the four variables that make up the proposed model have been developed. All the indicators that make up the latent variables are measured through 5-points Likert scale. Table 2 shows the definition and composition of the variables.

**Table 2:** Variables used in the research.

**Professionalization**	
	*In relation to the professionalization of internal processes in the company*, *please indicate your degree of agreement with the following statements*
Prof_1	There is a formally established organisational structure
Prof_2	There is a formalised staff performance and incentive system in place
Prof_3	There is an annual schedule and follow-up of management team meetings
**Risk-taking**	
	*In relation to the risk-taking propension*, *please indicate your level of agreement with the following statements*
Risk_1	I have a strong propensity for high-risk projects
Risk_2	I believe that knowing the environment, bold and far-reaching actions are necessary to achieve the company’s objectives
Risk_3	When faced with decision-making under conditions of uncertainty, I normally adopt a bold and aggressive stance in order to maximise the probability of exploiting potential opportunities.
**Technological innovation**	
	*The evolution of your company over the last two years*, *and compared to other companies in your sector*, *can be rated in relation to your company’s products and services*
Tech_1	The number of new products or services introduced by your company per year
Tech_2	The pioneering nature of your company’s introduction of new products or services
Tech_3	The speed of response to the introduction of new products or services by other companies in the sector
	*The evolution of your company during the last two years*, *and comparing it with the rest of the companies in your sector*, *can be qualified in relation to the following processes*
Tech_4	The number of process changes your company introduces per year
Tech_5	The pioneering nature of your company is introducing new processes
Tech_6	Speed of response to the introduction of new processes by other companies in the sector
**Performance**	
	*Please indicate your level of agreement with the following statements*, *taking the competition as a reference*
Perf_1	Offers higher quality products
Perf_2	Has more efficient internal processes
Perf_3	Has more satisfied customers
Perf_4	Adapts earlier to market changes
Perf_5	It is growing more
Perf_6	It is more profitable

Source: Authors

#### 2.2.1. Professionalization

In order to be able to measure the degree of professionalization of the companies, respondents were asked to answer from 1 (strongly disagree) to 5 (strongly agree) on the existence of an organisational structure, the existence of a system of incentives and employee performance and the frequency and scheduling of management meetings [8, 17, 67].

#### 2.2.2. Risk-taking

The risk-taking dimension has been created through the three items developed by Miller and Friesen [5]. For this purpose, respondents were asked to answer from 1 (strongly disagree) to 5 (strongly agree) about the propensity for high-risk projects, the relationship between the execution of courageous actions and business objectives, and the adoption of a courageous stance in situations of uncertainty in order to exploit potential opportunities.

#### 2.2.3. Technological innovation

According to Damanpour and Gopalakrishnan [68], two types of technological innovation have been identified: process innovation and product innovation. Consistent with previous studies [69, 70], six indicators have been used to create this variable. For this purpose, respondents were asked to answer from 1 (strongly disagree) to 5 (strongly agree) about the introduction of new products and services and on the implementation of new processes by the company.

#### 2.2.4. Performance

In order to measure the financial and non-financial performance of companies, a latent variable has been created, consisting of six indicators [6, 71]. For this purpose, respondents were asked to answer, concerning the competence, from 1 (strongly disagree) to 5 (strongly agree) about the quality of products, efficiency of internal processes, customer satisfaction, its ability to adapt to change and its growth and profitability.

## 3. Results

### 3.1. Data analysis

Employing Smart-PLS software 3.3 [72], this research has applied partial least squares equation modelling (PLS-SEM) [73]. As this model presents a mix of formative (professionalization) and reflective factors (risk-taking, technological innovation and performance), PLS consistent has been used [74]. In order to obtain standard errors and t-statistics to evaluate the model, a bootstrap method of resampling of 10,000 has been used [75].

We have chosen PLS-SEM to run our model because the model is formed by four composity type A [76]. In addition, PLS-SEM is an ideal technique when the relationships analysed are very complex, especially if there are mediating effects [77, 78], it does not require a very large sample size and the data does not have to be strictly standardised [79].

### 3.2. Overall model: Test of goodness-of-fit (GoF)

As our model has a confirmatory purpose, we have started the analysis of the estimated model by focusing on several rates of overall goodness of fit (Gof) established by Henseler [13]. The results are shown in Table 3.

**Table 3:** Test of model fit.

	Estimated Model		Saturated Model	
	Value	HI99	Value	HI99
SRMR	0.042	0.044	0.042	0.044
d_ULS_	0.302	0.332	0.302	0.329
d_G_	0.101	0.126	0.101	0.126

Standardized root mean square residual (SRMR). Unweighted least squares discrepancy (d_ULS_). Geodesic discrepancy (d_G_).

The outcome for the standardized root mean square residual (SRMR) is 0.042, which is considerably below the maximum limit of 0.08 established [13]. Moreover, various model fit analyses (SRMR, dULS, dG) have been carried out using bootstrap-based inference statistics. In Table 3 it can be seen how all the results are under the bootstrap-based 99% (HI99) percentile. Therefore, the discrepancy observed between the empirical correlation matrix and the one implied by the model is not significant. Hence, given the results obtained, it can be stated an excellent model fit [80].

### 3.3. Measurement model

This model is made up of one reflective construct (professionalization) and three formative constructs (risk-taking, technological innovation and performance). According to Hair et al. [66], the evaluation of the formative dimensions is not the same as for the reflective-type dimensions. The evaluation of the reliability and validity is not applicable in formative construct because they do not need to be correlated [81, 82].

For the reflective construct, in order to validate the measurement model, traditional measures of internal consistency, reliability and validity has been verified. For this reason, the factor loadings, Cronbach’s Alpha, composite reliability [83], the Dijstra-Henseler rho ratio [84] and the average variance extracted (AVE) has been analysed.

According to Valls Martínez et al. [85], these measures are determined as follows:

Composite reliability, which should range from 0.7 to 0.95, is the lower limit of internal consistency reliability of the reflective construct. This measure is determined by:

$$\rho_{c}=\frac{{\left(\sum_{k=1}^{K}l_{k}\right)}^{2}}{{\left(\sum_{k=1}^{K}l_{k}\right)}^{2}+\sum_{k=1}^{K}\text{var}(e_{k})}$$

where *l*_*k*_ is the outer loading of the manifest variable k corresponding to a latent variable measured with K indicators; *e*_*k*_ is the measurement error of k; and var(*e*_*k*_) corresponds to the measurement error variance and it is calculated as $1-l_{k}^{2}$.

Cronbach’s alpha is the upper limit of internal consistency reliability:

$$\text{Cronbach}’\text{s}\;\alpha =\frac{K\bar{r}}{1+(K-1)\bar{r}},$$

where $\bar{r}$ is the maean of the triangular correlation matrix.

The Dijkstra-Henseler’s Rho usually stans between the two previous measures [73]:

$$\rho_{A}≔{\left({\hat{w}}^{\prime }\hat{w}\right)}^{2}\frac{{\hat{w}}^{\prime }\left(S-\text{diag}\left(S\right)\right)\hat{w}}{´{\hat{w}}^{\prime }\left(\hat{w}{\hat{w}}^{\prime }-\text{diag}\left(\hat{w}{\hat{w}}^{\prime }\right)\right)\hat{w}},$$

where $\hat{w}$ is the estimated weight vector of the construct, and *S* is the empirical covariance matrix of the manifest variables.

The average variance extracted (AVE) is a measure of the convergent validity [86]:

$$\text{AVE}=\frac{\sum_{k=1}^{K}l_{k}^{2}}{K}.$$

It is considered acceptable when its value exceeds 0.5, which means that the construct explains more than 50% of its manifest variables variance.

The results of the tests carried out are shown in Table 4. All results exceed the minimum values established [87], except for the loading of one indicator, although its value close to 0.7 is acceptable, supporting the reliability and convergent validity for the construct and its dimensions.

**Table 4:** Measurement model results.

	**Mean**	**Loading**	**t-student** *	**Q** ^**2**^	**α**	**ρA**	**ρC**	**AVE**
**Professionalization**					0.761	0.764	0.762	0.516
**Prof_1**	4.019	0.673	10.7130					
**Prof_2**	2.994	0.757	12.9340					
**Prof_3**	3.516	0.723	11.6900					
	**Mean**	**Weights**	**t-student** *	**Q** ^**2**^	**VIF**			
**Risk-taking**				0.056				
**Risk_1**	2.077	0.411	10.020	0.063	1.525			
**Risk_2**	3.203	0.422	11.908	0.047	1.679			
**Risk_3**	3.023	0.367	9.732	0.058	1.669			
**Technological innovation**				0.159				
**Tech_1**	4.061	0.179	7.896	0.098	1.726			
**Tech_2**	3.526	0.265	9.692	0.206	1.660			
**Tech_3**	4.039	0.126	4.412	0.035	1.648			
**Tech_4**	3.742	0.237	10.512	0.171	1.833			
**Tech_5**	3.381	0.249	12.846	0.190	2.463			
**Tech_6**	3.152	0.254	11.240	0.198	2.396			
**Performance**				0.150				
**PERF_1**	3.200	0.168	8.953	0.091	1.592			
**PERF_2**	3.326	0.226	13.936	0.192	1.962			
**PERF_3**	3.094	0.211	13.361	0.133	2.170			
**PERF_4**	3.119	0.211	13.075	0.153	1.965			
**PERF_5**	3.129	0.251	16.299	0.233	2.608			
**PERF_6**	2.965	0.216	14.536	0.154	2.586			

Significance and standard deviations (SD) performed by 10,000 repetitions Bootstrapping procedure. Q_B_^2^: cross-validated redundancies index performed by a 9-step distance-blindfolding procedure. α: Chronbach’s alpha; ρ_A_: Dijkstra–Henseler’s composite reliability; ρ_C_: Jöreskog’s composite reliability; AVE: Average Variance Extracted; VIF: Variance Inflation Factor

*: All the loadings and weights are significant at a 0.001 level.

Source: Authors

For the formative constructs the significance and relevance of items as well as the Variance Inflation Factor (VIF) to exclude problems of collinearity, have been evaluated. This measure is determined by [85]:

$${\text{VIF}}_{k}=\frac{1}{1-R_{k}^{2}}.$$

The results in Table 4, show how all the weights are significant and VIF values are below 3, verifying the absence of collinearity issues [87].

Based on the results obtained for both types of constructs it can be stated that the model is well formed.

In addition, the predictive relevance of the model has been confirmed through a blindfolding procedure (omission distance of 9), where all the Q^2^ values are above 0 [88].

Finally, the satisfactory explanatory qualities of the model has been tested. For this purpose the predictive relevance of the exogenous construct has been evaluated. So, the Q_B_^2^ statistical test (a cross-validated redundancy index), has been carried out by the blindfolding method [89]. The results in Table 4 reveal that all Q_B_^2^ are greater than zero, confirming this quality [90].

### 3.4. Structural model

Before testing the hypotheses, the existence of any multicollinearity problem has been ruled out by analysing the variance inflation factor (VIF). As can be seen in Table 5, the VIF values are between 1 and 1.468. This means that these values are well below the recommended maximum limit of 3.3 [91] and therefore there are no multicollinearity problems in this model.

**Table 5:** Results of the hypothesis testing.

Structural paths	Path	t	f^2^	95CI		H	Supported
**Direct effects**					VIF		
Professionalization → Performance	0.269	3.365***	0.073	[0.146; 0.408]	1.419	H_1_	Yes
Professionalization → Risk-taking	0.329	4.960***	0.121	[0.220; 0.437]	1.000	H_2a_	Yes
Risk-taking → Performance	0.178	3.300***	0.038	[0.088; 0.267]	1.194	H_2b_	Yes
Professionalization → Technological innovation	0.451	6.785***	0.266	[0.344; 0.565]	1.121	H_3a_	Yes
Technological innovation → Performance	0.251	3.843**	0.062	[0.139; 0.355]	1.468	H_3b_	Yes
Risk-taking → Technological innovation	0.223	3.569***	0.065	[0.118; 0.322]	1.121	H_4a_	Yes
**Indirect effects**					VAF		
*Individual indirect effects*							
Professionalization → Risk-taking → Performance	0.059	2.792**		[0.027; 0.096]	12.854	H_2c_	Yes
Professionalization → Technological innovation → Performance	0.113	3.698***		[0.065; 0.166]	24.619	H_3c_	Yes
Risk-taking → Technological innovation → Performance	0.056	2.343*		[0.021; 0.099]	31.461	H_4b_	Yes
Professionalization → Risk-taking → Technological innovation	0.073	3.312***		[0.039; 0.110]	13.931	H_5_	Yes
Professionalization → Risk-taking → Technological innovation → Performance	0.018	2.308*		[0.007; 0.033]	3.922	H_6_	Yes
					VAF		
*Global indirect effects*							
Professionalization →Technological innovation	0.073	3.312***		[0.039; 0.110]	13.931		
Professionalization → Performance	0.190	4.705***		[0.125; 0.258]	41.394		
Risk-taking → Performance	0.056	2.343***		[0.021; 0.099]	31.461		
**Total effect**							
Professionalization → Technological innovation	0.524	9.051***					
Professionalization → Performance	0.459	6.977***					

R^2^ adjusted [99% CI in brackets]: Risk-taking: 0.105 [0. 450; 0.188]; Technological innovation: 0.315 [0.240; 0.414]; Performance: 0.296 [0.216; 0.413]. Blindfolding Q^2^ index as shown in Table 4; Standardized path values reported; f^2^: size effect index; 95CI: 95% Bias Corrected Confidence Interval; VIF: Inner model Variance Inflation Factors; VAF: Variance Accounted Formula x 100 represents the proportion mediated. Significance, t-Student, and 95% bias-corrected CIs were performed by 10,000 repetitions Bootstrapping procedure;

*: p < 0.05;

**: p < 0.01;

***: p < 0.001.

Only total effects that differ from direct effects are shown.

Source: Authors

According to Hair et al. [92], the structural model has been analysed through the study of the magnitude, significance, algebraic sign and the effect size index (f^2^) values of the standardized regression coefficients (path coefficients). Similarly, for the endogenous construct the determination coefficient (R^2^) values has been analysed. For this purpose, a bootstrap re-sampling for 10,000 subsamples has been carried out.

**Fig 2:**
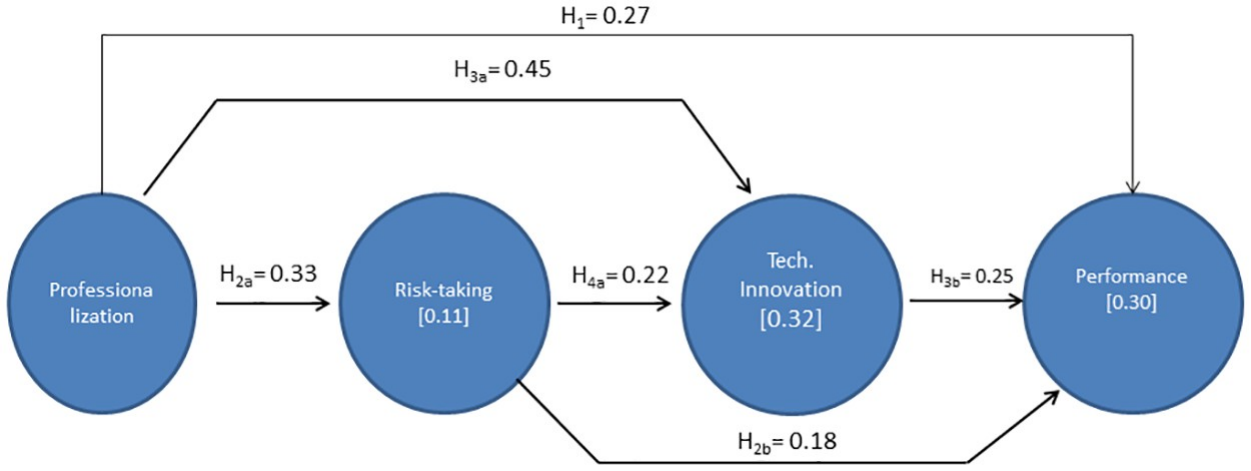
Results of SEM analysis.

The results in Table 5 and Fig 2 show that all hypothesis can be accepted. It can be observed that professionalizationsm has a significant positive effect on performance, risk-taking and technological innovation, with a path coefficent value of β = 0.269***, β = 0.329*** and β = 0.451*** respectively. Hence, these results verify the hypotheses H_1_, H_2a_ y H_3a_. Risk-taking has a significant positive effect on technological innovation and performance (β = 0.223*** and β = 0.178*** respectively),wich verifies H_2b_ and H_4a_. Finally, technological innovation has a significant positive effect on performance (β = 0.251***), verifyin H_3b_.

The R^2^ shows through the variables predicting an endogenous construct, how these can explain its variance. Therefore R^2^ is a measure of the predictive/explanatory power of the model [93]. Falk and Miller [94] established a minimun value of 0.10. The results obtained in this research show that the model explains 10,5% of the varience in risk-taking, 31,5% in technological innovation and 29,6% in performance, which demonstrates a moderate level of explanatory power for risk-taking and technological innovation.

The f^2^ analyse the contribution of the exogenous variables to the R^2^ of the endogenous variables. Values of 0.02, 0.15, and 0.35 indicate small, medium, and large effects [95]. As can be seen in Table 5, all links are above the minimum value of 0.02 [96], which shows its direct effect on the global performance variables. It is interesting to highlight the significant influence of professionalization on technological innovation (f^2^ = 0.266).

### 3.5. Mediation analysis

Once the direct effects have been analysed, following the procedure laid down by [79], indirect effects have been investigated. With the aim to check the indirect effects a bootstrapping procedure with 10,000 samples has been utilized [66]. This method generates for the individual indirect effect and the sequential mediation 95% bias-corrected. Additionally, the size of the indirect effect in relation to the total effect has been analyzed through the variance accounted for (VAF) [73]. The results are presented in Table 5.

The findings show that risk-taking mediates the impact of professionalization on technological innovation (β = 0.073***) and performance (β = 0.059***), supporting H_5_ and H_2c_. Similarly, it can be observed that technological innovation mediates the relationship between risk-taking and performance (β = 0.056*), supporting H_4b_, and between professionalization and performance (β = 0.113***), supporting H_3c_. Moreover, a sequential indirect effect of professionalization on performance through risk-taking and technological innovation has been verified (β = 0.018*), supporting H_6_.

According to Hair et al. [97], the variance accounted for (VAF) indicates the size of each of the indirect effects relative to the total effect. Therefore, the indirect effect of professionalization on technological innovation is about 13,9% of the total effect through risk-taking. Similarly, the indirect effect of professionalization on performance is about 41.39% of the total effect, with 12,85 through risk-taking, 24,6% through technological innovation, and an additional 3.92% sequentially. Finally, the indirect effect of risk-taking on performance is about 31.46% of the total effect through technological innovation. Since all effects are positive and the VAF values are below 0.8, it can be established that all mediations are partial and complementary [98].

## 4. Discussion and conclusion

This research aimed to analyse the effect of professionalization on firm performance, also examining the mediating effect of risk orientation and technological innovation. For this purpose, a sample of 310 Spanish SMEs has been used. The analysis was carried out using PLS-SEM.

The findings show, in line with previous research [7–9], that managers’ pursuit of their own interests means that the higher the level of professionalization of the company, the higher the performance obtained.

The results also reveal how professionalization enhances firms’ skills, allowing them to increase their Risk-taking capacity and thus better adapt to changes in the environment, which will increase their survivability and thus improve their performance. These results are in line with previous research.

On the other hand, and in line with previous research [1, 36], it has been shown that professionalization is key to being able to deal with appropriate technological innovation [43], as the latter requires the knowledge of the company. As technological innovation is a key factor in business performance, indirect professionalization affects performance by improving the innovative capacity of companies. Based on the above, it is also worth noting how it has been shown that professionalisation, by increasing the ability of companies to leave their comfort zone, indirectly influences technological innovation.

Furthermore, it has also been shown, in line with [56], that the greater capacity of companies to take risks will lead them to make greater investments aimed at increasing their technological innovation. Thus, risk orientation indirectly influences firm performance.

Finally, it is interesting to note how the results allow us to conclude the existence of a sequential influence of professionalization on risk orientation, the latter on technological innovation, resulting in a positive effect on performance.

The literature has repeatedly demonstrated the positive effect of professionalization on business performance [7–9]. Now this research shows how risk orientation and technological innovation increase the positive effect of professionalization on performance. The results show how companies that are more committed to professionalization obtain higher performance and that their impact will be greater as they improve their Risk-taking capacity and thus technological innovation.

From a theoretical point of view, this research contributes to the business management literature by integrating the role that risk orientation and technological innovation play in the relationship between professionalization and performance. This is of vital importance to understand what strategies SMEs can develop to survive and grow in today’s changing environment.

This research has important implications for SME owners and shareholders. It has been demonstrated that a commitment to professionalization will increase company performance through the ability to take risks and carry out technological innovations to develop new products and services. Likewise, our results have shown that in order to innovate, companies have to leave their comfort zone and take risks, which undoubtedly requires prior skills that cannot be obtained without a process of business professionalisation.

This research is not without limitations, which may serve as a basis for future lines of research. This study was carried out only with Spanish SMEs, so that the results may not be extrapolated to other geographical areas. Future research could cover a more ambitious sample of SMEs from several countries. Also, this research only uses cross-sectional data, so that the results may change over time. It would be interesting for future research to use longitudinal data to analyse the effects over time.
